# Malignant mesothelioma of the tunica vaginalis of the testis without exposure to asbestos

**DOI:** 10.1186/1757-1626-1-310

**Published:** 2008-11-14

**Authors:** Ashish Goel, Akansha Agrawal, Rajiv Gupta, Smriti Hari, A B Dey

**Affiliations:** 1 Senior Research Associate, Medicine, AIIMS, New Delhi 110029, India; 2 Junior Resident, Medicine, AIIMS, New Delhi, 110029, India; 3 Associate Professor, Medicine, AIIMS, New Delhi 110029, India; 4 Assistant Professor, Radiology, AIIMS, New Delhi 110029, India; 5 Professor, Medicine, AIIMS, New Delhi 110029, India

## Abstract

**Introduction:**

Mesotheliomas are rare tumours that usually are seen in the pleura after asbestos exposure. Mesotheliomas have been reported around the testicular region but are even rarer following trauma, herniorrhaphy and long term hydrocoele.

**Case presentation:**

An elderly male farmer presented to us with an insidious onset of painless swelling in his left lower limb which gradually progressive. At the time of presentation it had involved his entire limb. A hard palpable mass of size 5 * 4 cms was detected in the left iliac fossa and a testicular enlargement was noted on the left side. The ultrasound of the testes showed that the left testis was enlarged 3.9*3*3.2 cms showing diffusely heterogenous echo-texture and irregular nodular surface with irregular hypoechoic thickening of the scrotal wall with left sided hydrocele. A separate hypoechoic *1.2 cms lesion was visualized in the anterior scrotal wall. FNAC from the scrotal mass showed tumour cells of simialr morphology present singly in monolayered sheets and in three dimensional fragments. The overall immunomorphological features suggested a malignant mesothelioma likely to have arisen from the tunica vaginalis.

**Conclusion:**

In conclusion, though a rare tumor, malignant mesothelioma of the tunica vaginalis of the testis should be considered whenever a paratesticular mass lesion is seen even without a history of trauma or asbestos exposure as is highlighted in this case. Ultrasound findings are helpful and fine needle aspiration of the tumor may assist in arrival at a diagnosis. Surgical orchidectomy remains the modality of treatment.

## Introduction

Mesotheliomas are uncommon tumours that occur in the pleura or peritoneum usually following asbestos exposure which may be of any duration and sometimes several years in the past. They are not related to smoking and those originating in the tunica vaginalis of testis are rare, representing less than 5% of all mesothelioma [1]. Trauma, herniorrhaphy and long term hydrocele are other possible predisposing factors. Other mesothelial lesions involving the paratesticular region include mesothelial cysts, reactive mesothelial hyperplasia, adenomatoid tumors, benign cystic mesothelioma, well-differentiated papillary mesothelioma, and malignant mesothelioma.

## Case presentation

A 65-year-old ambulatory male farmer from Bihar presented to our outpatient department with painless swelling of his left leg of two months that was insidious in onset and gradually progressive. The swelling started from his thigh and now involved the entire limb. He had no identifiable risk factors for a deep vein thrombosis. He had no bowel or bladder symptoms. The patient did not report any previous surgery or trauma to the region. He had worked as a hired farm worker all his life and gave no history of exposure to asbestos in his occupation.

On examination he had stable vitals, and had pitting edema of the left leg. His right lower leg was normal. The ankle and knee joint were normal. No ulcers were seen on his leg. A difference in measurement between the two limbs of 5 inches over the thigh and 4 inches over the calf was noticed. He had inguinal lymphadenopathy on the left side, but no nodes were apparent to palpation on the right.

A diagnosis of deep vein thrombosis and elephantiasis were entertained at initially. An examination of the abdomen revealed a hard palpable mass of size 5 * 4 cms in the left iliac fossa. This was a hard globular smooth nontender mass with lower margins disappearing below the inguinal ligament. On leg raising test the lump was found to decrease in prominence. The spermatic cord was palpated as a hard and cord like structure. The scrotal skin was normal. The left testis was found to be enlarged as a single hard mass of 10*5*4 cms. The testis was not tender and testicular sensation was preserved. The swelling was not fluctuant and not trans-illuminant. Per rectal examination did not reveal any abnormality.

The initial diagnosis was now revised to suspect a testicular tumor – probably a seminoma or a lymphoma of the testes.

The initial investigations revealed normal blood picture with a hemoglobin of 13 g/dL, a slight leukocytosis of 11200/cmm with neutrophilic predominance. He had normal renal and liver functions and the electrolytes were found normal. No abnormality was revealed on the chest radiograph and the color Doppler of the lower limb showed no evidence of deep vein thrombosis.

The echocardiograph of the patient showed normal rhythm and no atrio-ventricular blocks were identified. The ultrasound examination of the abdomen and scrotum showed multiple well defined hypoechoic lymph nodes in the pre and para aortic regions along the left common iliac vessels. Right testis measured 3.2*2.1*2.4 cms and a right sided hydrocele was noticed. The left testis was enlarged 3.9*3*3.2 cms showing diffusely heterogenous echo-texture and irregular nodular surface with irregular hypoechoic thickening of the scrotal wall with left sided hydrocele. A separate hypoechoic lesion was visualized in the anterior scrotal wall. There was left sided hydrocele. The epididymis was normally visualized. The prostate was normal with a size of 2*3.5*2.7 cms.

US guided FNAC from the left iliac mass were performed which revealed cells suggestive of a malignancy but were inconclusive for characterization.

Computerized tomographic scan of the abdomen and the chest revealed few fibrotic lesions in Right Upper and apical segments of the right lower lobes of the lung. Multiple conglomerate lymphnodes (largest 5*4 cms) were seen in the retroperitoneal and pre pancreatic locations. A large (9*7*7 cm) lymph nodal mass was seen in the left hemi pelvis encasing left common iliac and left external and left internal iliac vessels. Pelvic and superficial inguinal lymphnodes (~2 cm) were also found to be enlarged. A simple cyst was visualized in the left kidney

Alpha fetoprotein was found to be 4.64 ng/ml (normal < 10) and beta human chorionic gonadotropin level was found to be 0.29 mIU/ml (normal < 4)

Trucut biopsy of the left iliac mass showed features of a squamous cell carcinoma. However, this was not consistent with the final diagnosis achieved after immunomorphological studies.

Fine needle aspiration cytology from the scrotal mass was performed which showed tumour cells of similar morphology present singly in monolayered sheets and in three dimensional fragments. The tumour cells were polygonal in appearance with abundant cytoplasm and nuclei with vesicular chromatin, mild pleomorphism and eosinophilic nucleoli. The tumour cells were found positive for cytokeratin, epithelial membrane antigen (EMA) and calretinin. The overall immunomorphological features suggested a malignant mesothelioma likely to have arisen from the tunica vaginalis. The cytopathological findings of this case have been reported in detail by Mathur et al [2].

## Discussion

Mesotheliomas of the paratesticular region usually present as tumors with no specific findings, have a broad age of distribution making pre-operative diagnosis difficult [3,4]. Although specific findings are not seen, but long lasting hydrocele has been reported as a predisposing factor [5]. Ultrasound features show hydrocele which may be associated with well organized tissue fronds of mixed echogenicity; or multiple extratesticular nodular masses of increased echogenicity originating from the scrotal wall; or focal thickening of tunica vaginalis with nodularity; and lobulated mass lesions occupying the left epididymal head mimicking an epididymal tumor [6-9]. Although mesotheliomas have been known to secrete markers calretinin, mesothelin [6,10], CAM 5.2 [11], Alcian blue [12], Hale colloidal iron [12], vimentin [1,12], cytokeratin [1,12], calretinin [1], HBME-1 antigen [1], endothelial cell markers QBend-10 (CD 34), Factor VIII-related antigen (vWF) and UEA-1, panepithelial antibody (Lu-5) and membranous staining for BMA-120. [13] yet the diagnosis may remain elusive on fine needle aspiration cytology in the pre-operative stage. The tumor has been found to be negative for CEA [11,12], Factor VIII related antigen [12], Ber-EP4, HEA-125 and blood group related antigens A, B and H [13]. Reports for other markers such as Leu M1 and EMA remain conflicting and contradictory [11-13].

Mesotheliomas are difficult to manage and no clear guidelines exist for management. Surgery has been suggested and orchidectomy is generally performed. Patients have been reported to have remained tumor free at 6 years following inguinal orchidectomy[14] for limited tumor and at 2 years with lymph node metastasis.

## Conclusion

In conclusion, though a rare tumor, malignant mesothelioma of the tunica vaginalis of the testis should be considered whenever a paratesticular mass lesion is seen even without a history of trauma or asbestos exposure as is highlighted in this case. Testicular examination is usually neglected in medical departments while examining the abdomen and this oversight usually gives erroneous and misleading diagnosis. It is known that findings missed during the initial patient examination, remain mysteriously elusive during the further course of treatment. Ultrasound findings are helpful and fine needle aspiration of the tumor may assist in arrival at a diagnosis. Surgical orchidectomy remains the modality of treatment.

## Consent

Written informed consent for publication of this case report could not be obtained from the patient or from their next of kin. The patient underwent a high inguinal orchidectomy and then was lost to follow-up. Extensive attempts made to trace the patient proved futile. We believe this case report holds worthwhile clinical information which could not be communicated as effectively in any other way. We would not expect the patient or the family to object to publication.

## Competing interests

Since the time of submission it has come to the notice of the authors that the detailed cytopathological findings of the same case have been submitted by a separate set of authors in another publication[2].

## Authors' contributions

AG wrote the first draft of the manuscript. AA was involved in day to day patient care. RG provided insight and valuable inputs into the manuscript. SH delineated the radiological findings of the case. ABD was responsible for the overall care of the patient and the final decision on the patient management rested with him.
